# Right Compared With Left Thoracic Approach Esophagectomy for Patients With Middle Esophageal Squamous Cell Carcinoma

**DOI:** 10.3389/fonc.2020.536842

**Published:** 2020-10-26

**Authors:** Yan Zheng, Yin Li, Xianben Liu, Ruixiang Zhang, Haibo Sun, Wenqun Xing

**Affiliations:** ^1^Department of Thoracic Surgery, The Affiliated Cancer Hospital of Zhengzhou University, Henan Cancer Hospital, Zhengzhou, China; ^2^Department of Thoracic Surgery, National Cancer Center/National Clinical Research Center for Cancer/Cancer Hospital, Chinese Academy of Medical Sciences and Peking Union Medical College, Beijing, China

**Keywords:** esophageal cancer, esophagectomy, survival analysis, squamous cell carcinama, surgery

## Abstract

**Background:** In China, open surgical approaches for esophageal cancer (EC) can be divided into two techniques, the right- and left- transthoracic esophagectomy. Although there is an increasing number of instances that use the right side, the optimal surgical technique remains unclear. Based in a large cancer center with rich experience of both transthoracic side approaches, this study compared the long-term survival of patients treated by these two surgical techniques.

**Methods:** The patients included in this study underwent a right transthoracic esophagectomy (Right, McKeown) or left transthoracic esophagectomy (Left, Sweet, or chest neck dual-incision) for esophageal squamous cell carcinoma (ESCC) between January 2015 and October 2018. The overall survival(OS) rate and perioperative data between the two groups were then retrospectively analyzed.

**Results:** We included 437 patients who underwent Right (*n* = 202) and Left (*n* = 235) approaches for ESCC. There was a significantly longer median operative time (250 vs. 190 min, *P* < 0.001) and longer median postoperative hospital stay (17 vs. 14 days, *P* < 0.001) in the Right side group. The OS at 5-years was 49.9% in the Right group and 52.45% in the Left group; hazard ratio (HR) (95% CI): 1.002 (0.752–1.337), *p* = 0.987.

**Conclusions:** For middle thoracic ESCC without suspected lymph node metastasis in the upper mediastinum, the esophagectomy through the Left thoracic approach could achieve the same OS as the Right side, with better short-term outcomes.

## Introduction

Esophageal cancer (EC) is ranked as the sixth most common cause of cancer-related death in the world (1). Surgery is considered the best choice for EC, but in China, there is still a lack of consensus regarding the use of left and right thoracic transthoracic approaches, and debates have become increasingly complex over the past 5 years (2–6). This reflects previous debates in western countries regarding the transhiatal vs. transthoracic approach for EC (7, 8). This was resolved by a randomized control trial (RCT) that demonstrated there was no significant difference in overall survival (OS) between transhiatal and transthoracic approaches for EC (7, 8). However, an RCT by Dr. Chen et al. in China concluded that the right transthoracic approach was better for patients with increased OS in esophageal squamous cell carcinoma (ESCC) (3). The study indicated that the extended radial lymphadenectomy of the right transthoracic approach has benefits in terms of survival (3). Despite these findings, a domestic online survey showed that only 27.8% of EC patients received an esophagectomy through the right thoracic approach in China in 2012 (9). Die to these RCT results and the advocation of the Chinese Anti-Cancer Association (9), the number of instances were the right thoracic approach is used is dramatically increasing in China. However, the left transthoracic approach has merits in that there is a lower risk of postoperative complications, shorter operation time, and faster recovery time (6, 10). Therefore, we performed a retrospective study, using a prospective database to compare right and left approaches to esophagectomy for middle thoracic ESCC conducted in a large-scale cancer center with extensive experience of the left thoracic approach.

## Patients and Methods

### Patients

This study was approved by the ethics review committee of the Affiliated Cancer Hospital of ZhengZhou University Henan Cancer Hospital (approval number 2018138). The Thoracic Surgery Department of Henan Cancer Hospital has created a prospective database of the department with the help of the LinkDoc company. The details of patients were collected on the 1st day of hospitalization and included pretreatment examinations, treatment, and follow-up data.

The inclusion criteria of this study were: 1. consecutive patients with thoracic ESCC from January 1, 2015, to October 7, 2018; pathological T stage 1b-4 according to the 2009 American Joint Committee on Cancer (AJCC) TNM staging system; open transthoracic esophagectomy; and middle thoracic ESCC. We excluded patients for whom follow-up information was missing. They were divided into two groups, the Right group (McKeown) and the Left group (Sweet or chest neck dual-incision).

Each patient finished preoperational tests, including electronic ultrasound gastroscopy with a pathological examination, contrast thoracic and upper abdominal CT scanning, upper gastrointestinal contrast imaging, abdominal and cervical color ultrasound, emission computed tomography (ECT), pulmonary function test, electrocardiography, and other routine tests. If the positron emission tomography-CT (PET/CT) was accepted by patients, it was adopted. In total, <10% of patients accepted the PET/CT.

The surgical procedures were conducted by 10 surgeons in total, each of whom had extensive surgical experience in transthoracic esophagectomies on both the left and right sides. If there were no suspected positive lymph nodes in the superior mediastinum. The selection of the right or left approach was dependent on the informed choice of each surgeon. The definition of postoperative complications is outlined in Supplementary Material 1.

### Surgical Procedures

Following the McKeown method for the Right approach to the procedure, the patient was initially placed in the left lateral decubitus, an incision was then made in the fourth or fifth intercostal space, the azygos vein was dissected, and the esophagus was mobilized. If the thoracic duct was injured it was removed, otherwise, it was preserved. Next, the patient was positioned in the supine position. An upper midline abdominal incision was made in the stomach, mobilized, and the left gastric artery was resected. A gastric conduit was constructed using linear staplers (EC60, Ethicon, Cincinnati, USA). The esophagus was then resected in the neck. The gastric tube was delivered through the thoracic cavity to the left side of the neck and a mechanical or hand-sewn cervical esophagogastric anastomosis was adopted.

In the Left approach to the procedure, a left-sided thoracic incision was made at the sixth or seventh intercostal space. The esophagus was then mobilized and resected. To access the abdominal cavity, the diaphragm was incised, the stomach mobilized, and a gastric tube was made. The residual stomach with the esophagus was removed. Finally, the gastric tube was delivered to the left side of the neck incision. A mechanical or hand-sewn cervical esophagogastric anastomosis was conducted.

In the right thoracic procedure, a total mediastinal lymphadenectomy was used. The bilateral recurrent nerve lymph nodes were resected. Except for the recurrent nerve lymph nodes, the two procedures could acquire the same lymphadenectomy. The middle and lower periesophageal, subcarinal, lower posterior mediastinum, perigastric, common hepatic, celiac arteries, and the left gastric artery lymph nodes were removed.

### Follow-Up

After surgery, follow-up surveillance tests were every 3 months in the first 2 years, 6 months between 3 and 5 years, and every year after 5 years. The chest CT scans and abdominal/cervical color ultrasound were routinely tested. If a patient had special symptoms, they may have received other tests. OS was defined as the duration from the date of surgery to death.

### Statistical Analysis

The Mann-Whitney *U*-test and the chi-square test were used to evaluate the association between the two groups in the clinicopathological variables. Kaplan-Meier curves were employed to analyze the OS. A multivariate analysis of survival was conducted using the Cox proportional hazards regression model. Covariates with clinical value and those factors with a *P* ≤ 0.2 in the univariate analysis were included in the multivariate model. R language 3.4.1 for Windows was used to fulfill the statistical analysis. A value of *P* < 0.05 was considered statistically significant.

## Results

From January 1, 2015, to October 7, 2018, a total of 437 patients met the inclusion criteria and were included in the study. There were 202 (46.22%) patients in the Right group (McKeown) and 235 (53.78%) patients in the Left group (Sweet or chest neck dual-incision). The clinical characteristics of patients in the two groups are listed in Table 1. Most of the patients in the study were male. The number of female participants, as well as age, BMI, smoking history, drinking history, and clinical N stage, were slightly higher in the left group than in the right group. The right group included a higher proportion of patients from the rural cooperative medical care system. A higher proportion of patients in the right group (17.82%), compared to in left group (15.31%), accepted neoadjuvant treatment, while a higher proportion of patients in the left group (52.34%), compared to in right group (47.29%), received adjuvant treatment. There was no statistically significant difference in the clinical characteristics of the two groups (Table 1).

**Table 1:** Baseline demograpic and clinical characteristics of patients of the entire cohort.

**Variable**	**Caese (*N* = 437)**	**Surgical approaches (%)**		***P*-value**
		**Right (*N* = 202)**	**Left (*N* = 235)**	
**Mean Age, median (range)**	437 (100%)	63.5 (43–77)	65 (41–81)	0.136
**Age** ***N*** **(%)**				0.400
≥64	228 (52.17%)	101 (50.00)	127 (54.04)	
<64	209 (47.83%)	101 (50.00)	108 (45.96)	
**Mean BMI, mean (*****SD*****)**	437 (100%)	23.23 (3.03)	23.65 (3.02)	0.204
**Sex** ***N*** **(%)**				0.323
Female	138 (31.58)	59 (29.21)	79 (33.62)	
Male	299 (68.42)	143 (70.79)	156 (66.38)	
**Smoking** ***N*** **(%) (3 missing data)**				0.597
Never	224 (51.61)	101 (50.25)	123 (52.79)	
Ever/current	210 (48.39)	100 (49.75)	110 (47.21)	
**Drinking** ***N*** **(%) (5 missing data)**				0.942
Never	243 (56.25)	111 (56.06)	132 (56.41)	
Ever/current	189 (43.75)	87 (43.94)	102 (43.59)	
**Medical insurance** ***N*** **(%) (2 missing data)**				0.464
Rural cooperative medical care system	265 (61.43)	127 (63.18)	138 (59.74)	
Others (city residents and works, self-paying,	167 (38.66)	74 (36.82)	93 (40.26)	
social insurance, free healthcare, others)				
**cT stage** ***N*** **(%)**				0.601
T1b-2	134 (30.66)	58 (28.71)	76 (32.34)	
T3	268 (61.33)	129 (63.86)	139 (59.15)	
T4	35 (8.01)	15 (7.43)	20 (8.51)	
**cN stage** ***N*** **(%)**				0.828
N0	321 (73.46)	147 (72.77)	174 (74.04)	
N+	116 (26.54)	55 (27.23)	61 (25.96)	
**Neoadjuvant treatment** ***N*** **(%)**				0.519
Yes	72 (16.48)	36 (17.82)	36 (15.32)	
No	365 (83.52)	166 (82.18)	199 (84.68)	
**Adjuvant treatment** ***N*** **(%)**				0.338
Yes	219 (50.11)	96 (47.29)	123 (52.34)	
No	218 (49.98)	106 (52.22)	112 (47.66)	

*MIE, minimally invasive esophagectomy; OE, open esophagectomy; N, number; SD, standard deviation; cT, clinical tumor; cN, clinical lymph nodes*.

Intraoperative and postoperative data are shown in Table 2. The median operation time was 250 min in the right group and 190 min in the left group (*P* < 0.001). During the operation, the mean blood loss was 236.61 ml in the right group and 220.54 ml in the left group (*P* = 0.708). The median lymph nodes retrieved were four higher in the right group than in the left group (*P* = 0.002). The level of anastomosis for the right and left group was comparable (cervical/thoracic: 201/0 vs. 221/9, *P* = 0.8). No patients died during their postoperative hospital stay nor the first 90 days after the operation. The patients in the left group had a significantly shorter postoperation hospital stay than the right group (median number of days: 14 vs. 17, *P* < 0.001). The anastomotic leakage rate in the right group was 1.98%, vs. 2.13% in the left group, without a statistically significant difference (*P* > 0.999). The pathological data between the two groups were without significant difference (*p* > 0.3) (Table 2).

**Table 2:** Intraoperative and Postoperative characteristics of the two groups.

	**Surgical approaches (%)**		***P*-value**
	**Right (*N* = 202)**	**Left (*N* = 235)**	
**Intraoperative Data**			
Median operative time (min)	250	190	<0.001^*^
Mean operative time (*SD*) (min)	293.02 (314.94)	201.77 (48.76)	<0.001^*^
Mean blood loss (*SD*) (mL)	236.61 (239.18)	220.54 (189.23)	0.708
Median lymph nodes retrieved (range) *N*	25 (8–60)	21 (8–54)	0.002^*^
R1/R2 resection *N* (%)	2(1.04)	0	0.238
**Level of anastomosis** ***N*** **(%)**			0.800
Cervical	201 (100)	221 (96.09)	
Thoracic	0 (0)	9 (3.91)	
**Anastomosis method** ***N*** **(%)**			0.001^*^
Manual anastomosis	85 (42.08)	123 (52.34)	
Mechanical anastomosis	105 (51.98)	111 (47.23)	
Semi-mechanical anastomosis	12 (5.94)	1 (0.43)	
**Postoperative data**			
Median postoperative hospital stay days (range)	17 (7–68)	14 (4–75)	<0.001^*^
Median mediastinal tube drainage days (range)	7 (3–34)	7 (3–28)	0.175
Myocardial arrhythmia *N* (%)	20 (9.90)	35 (14.89)	0.117
Pneumonia *N* (%)	34 (16.83)	26 (11.06)	0.081
Anastomotic leakage *N* (%)	4 (1.98)	5 (2.13)	>0.999
In hospital mortality/90-days mortality *N* (%)	0	0	NA
**Pathological data**			
**pT stage** ***T*** **(%)**			0.950
T1b	9 (4.46)	23 (9.79)	
T2	56 (27.72)	46 (19.57)	
T3	112 (55.45)	138 (58.72)	
T4	25 (12.38)	28 (11.91)	
**pN stage** ***N*** **(%)**			0.546
N0	111 (54.95)	143 (60.85)	
N1	56 (27.72)	61 (25.96)	
N2	27 (13.37)	25 (10.64)	
N3	8 (3.96)	6 (2.55)	
**pTNM staging 7th** ***N*** **(%)**			0.398
IA	6 (3.02)	11 (4.85)	
IB	33 (16.58)	43 (18.94)	
IIA	31 (15.58)	28 (12.33)	
IIB	40 (20.10)	52 (22.91)	
IIIA	40 (20.10)	43 (18.94)	
IIIB	17 (8.54)	17 (7.49)	
IIIC	32 (16.08)	33 (14.54)	

*MIE, minimally invasive esophagectomy; OE, open esophagectomy; N, number; SD, standard deviation; NA, Not Available; pN, pathological lymph nodes; pTNM, tumor/node/metastasis*.

* *Statistically significant (p < 0.05)*.

**Figure 1:**
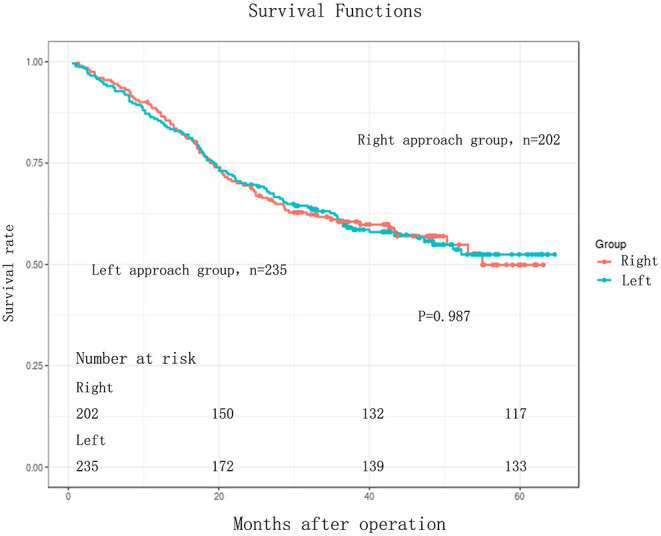
Kaplan-Meier curves for comparison of overall survival between Right and Left in EC patients (*n* = 437). Overall survival (OS) between the two groups were without any significant difference (log-rank test, *P* = 0.987).

The follow-up period ranged from 3 to 64.60 months. The median follow-up period was 33 months. For the whole cohort, the 5-years OS rate was 51.44% (95% CI: 45.86–57.71). The 5-years OS rate for the right group was 49.90% (95% CI: 40.90–60.87) and for the left group, it was 52.45% (95% CI: 45.64–60.27). There was no statistically significant difference between the right and left groups, *p* = 0.987 (Figure 1, Table 3). This conclusion is consistent with data produced by the multivariable Cox regression model (Table 3).

**Table 3:** Univariate and multivariate analyses of overall survival in 437 esophageal carcinoma patients.

**Variables**	**Univariate analysis**			**Multivariate analysis**		
	**HR**	**95%CI**	***P*-value**	**HR**	**95%CI**	***P*-value**
Age (<64 vs. ≥64 years)	0.817	0.611–1.092	0.171	0.782	1.581–1.051	0.103
Gender (Male vs. Female)	0.944	0.696–1.280	0.712	0.756	0.502–1.137	0.179
Smoking (Yes vs. No) (3 missing data)	1.190	0.893–1.586	0.235	1.181	0.710–1.967	0.522
Alcohol (Yes vs. No) (5 missing data)	1.151	0.863–1.535	0.338	1.227	0.768–1.960	0.391
Medical insurance (Rural vs. Others)	1.110	0.822–1.498	0.495			
Blood loss (≤ 200 vs. >200 ml)	0.840	0.565–1.294	0.390			
Operation time (≤ 180 vs. >180 min)	0.821	0.580–1.162	0.265			
Lymph nodes retrieved (≤ 23 vs. >23)	1.112	0.834–1.484	0.469			
pT stage (T1–2 vs. T3–4)	2.059	1.431–2.963	0.0001			
pN stage (N0 vs. N1–3)	2.570	1.917–3.446	<0.0001^*^			
pTNM staging 7th (I, II vs. III)	6.438	0.327–0.587	<0.0001^*^	0.431	0.321–0.579	<0.001^*^
Surgical approach (R vs. L)	1.002	0.752–1.337	0.987	1.031	0.771–1.377	0.838

*HR, Hazard Ratio; CI, confidence interval; pN, pathological lymph nodes; pTNM, pathological tumor/node/metastasis; R, minimally invasive esophagectomy; L, open esophagectomy*.

* *Statistically significant (p < 0.05)*.

## Discussion

This retrospective study compared the long-term OS of two widely adopted surgical approaches for resectable thoracic ESCC in China. The data showed no difference in the 5-years OS of two groups of patients without suspected upper mediastinal lymph node metastasis in preoperation tests.

In China over the past 5 years, there has been discussion as to whether the left or right transthoracic approaches were better. In 2013, a national survey showed that only 27.8% of ESCC received the right transthoracic approach (9). However, after it was advocated by the Chinese Anti-Cancer Association (9), the use of the right approach dramatically increased. Henan Cancer Hospital is located in the highest incidence area of ESCC in China and is the largest cancer center in China, and the proportion of operations that have used the right approach has increased in recent years. In the Linkdoc database of our department, before January 1, 2015, the proportion of operations that used the right approach was 8.13%. In 2015, the proportional use of the right approach increased to 66.90%, and in 2017, the right approach accounted for 81.82%. Some studies have argued that the survival benefits of the right approach are because it involves a more radical lymphadenectomy of upper mediastinal lymph nodes. This was demonstrated by the RCT of Dr. Haiquan Chen et al. (3) in data that showed that the right approach is associated with increased OS in ESCC, particularly in those with lymph node involvement (HR, 0.632; 95% CI, 0.412–0.969, *P* = 0.034) and/or R1–2 resection margins (HR, 0.495; 95% CI, 0.290–0.848, *P* = 0.009) (2). As discussed here, the practices of Chinese thoracic surgeons have changed in response to this study, but the study itself is based on limited evidence.

In the long-term analysis by Chen (2, 3), the 3-years OS rates were 74 and 60% for the right and left approaches, respectively (HR, 0.663; 95% CI, 0.457–0.961; *P* = 0.029) (2). However, this trial has limitations, as the short-term analysis outlines that the mean operation time for the Sweet group was only 30 min faster than the Ivor-Lewis group, which is an unacceptable difference for most cancer centers with experience of the left approach procedure (10, 11). In contrast, the difference in median operation time in our left/right cohort reached 60 min. The difference in mean operation time reached 91.25 min. In the study by Chen, the median hospital stays for the Sweet group were 2 days (*p* = 0.002) longer than the Ivor-Lewis group, which is also unusual based on data from past studies (6, 10, 11). What is more, the total complications in the Sweet group were even higher than in the Ivor-Lewis group, 62 vs. 45% (*p* = 0.04) (2). This indicates that Chen et al. based their assessment of the benefits of the left approach from short-term data. The left approach has benefits which include a shorter operation time, fast recovery, fewer complications, and shorter postoperation stay (6, 10, 11), which accounted for the popularity of the Sweet procedure in China before 2013 (9). However, the data in the study by Chen does not reflect the short-term benefits of the left approach. Although it was an RCT, the results of the study by Chen should be carefully interpreted, especially in terms of the short-term results, which are different from those established by other studies (6, 10, 11). Except for the differences in approach to lymphanectomy of the left and right approaches, the surgical incision itself may affect the survival. With the same extent of lymphanectomy, another retrospective study has demonstrated that the MIE could achieve lower operative morbidity and long time survival benefits (12). The survival benefits may due to the different incisions.

The advocation of the survival benefits of the right side approach were mainly based on the radical resection of upper mediastinal lymph nodes (3, 9). The metastases rate of upper mediastinal lymph nodes was around 30% (13). The patients without suspected upper mediastinal lymph nodes, the negative predictive value of preoperative chest CT scan was 99.23% (14). Maybe some patients do not need superior mediastinal lymphanectomy.

The lymph nodes metastases of ESCC is an important prognostic factor. The number of positive lymph nodes (15), the ratio of positive lymph nodes (16), the number of total resected lymph nodes (17), have been suggested to have prognostic value. The radical lymphanectomy also has diagnostic value and more precise pathological stage classification to indicate the adjuvant treatment (18, 19). These may all contribute to OS benefits.

The treatment value of lymphanectomy is also still under debate (20–23). Hsu et al. have demonstrated that although 30% of ESCC had positive results for right upper mediastinal lymph nodes, there were no significant differences in survival rates between patients with or without lymphadenectomy of the right upper mediastinal (24), indicating that the lymph node dissection itself might not have benefits in terms of survival. It is therefore not appropriate to mix the different incisions with the different extents of lymphanectomy when discussing how these factors might influence survival. The left approach (Sweet or chest neck dual-incision) has been in existence for ~80 years in China (25). It was the most used method chosen by thoracic surgeons before 2013 (9). One needs to be cautious when saying it has a better/worse impact on ESCC, especially for clinically negative lymph nodes of the upper mediastinum. This concept is also supported by Yang Ding et al. They found that for the middle and lower thoracic EC patients, with or without clinical lymph node metastasis, the surgical treatment through the right thoracic approach can achieve the same OS as the left thoracic approach (5).

The data used in this study was gathered from a prospective database. Our cancer center is a high-volume cancer hospital, located in an area with the highest incidences of EC worldwide, and the thoracic surgeons working there have a large amount of experience in using the left approach. Because the pretreatment parameters of the two groups in our study were without significant difference, the confounding biases of this study were well-controlled. However, this study did still have some limitations, particularly connected to the fact that it was a retrospective study. The LinkDoc data company was employed by our department to manage the database from 2015, meaning that long-term follow-ups still need further evaluation. At the time of publication, the information on the database including data on recurrence and postoperation complications were still under construction. Data on disease-free survival, local recurrence, and distant recurrence could not be analyzed as part of this study and we were not able to perform the Clavien-Dindo classification of postoperation complications.

The long-term oncological differences between the right and left approaches still need to be evaluated by a well-designed multicenter RCT in the future, and any changes in clinical practice should be based on further high-level evidence.

## Data Availability Statement

The datasets analyzed in this article are not publicly available. Requests to access the datasets should be directed to the ethics review committee of the Affiliated Cancer Hospital of ZhengZhou University/Henan Cancer Hospital.

## Ethics Statement

The studies involving human participants were reviewed and approved by The ethics review committee of the Affiliated Cancer Hospital of ZhengZhou University/Henan Cancer Hospital approved this study (Number. 2018134). The patients/participants provided their written informed consent to participate in this study.

## Author Contributions

YZ and WX: conceived and designed the study. YZ, YL, XL, and RZ: performed the experiments. YZ and HS: analyzed the data, contributed reagents, materials, analysis tools, and wrote the paper. All authors contributed to the article and approved the submitted version.

## Conflict of Interest

The authors declare that the research was conducted in the absence of any commercial or financial relationships that could be construed as a potential conflict of interest.
