# Supplementary material for: Right Compared With Left Thoracic Approach Esophagectomy for Patients With Middle Esophageal Squamous Cell Carcinoma
Source: Front Oncol. 2020 Oct 26;10:536842. doi: 10.3389/fonc.2020.536842 (PMC7649421; doi:10.3389/fonc.2020.536842)
Supplement: Supplementary file 1 [file Data_Sheet_1.pdf]

Additional file 1: Definition of complications

Complication	Criteria
Cardiac Complications	
Cardiac infarction	Confirmed by electrocardiography or echocardiography and cardiac enzyme monitoring
Heart failure	Confirmed by echocardiography or necessitating pressure agents
Arrhythmia	ECG confirmed and necessitating medication
Pericarditis	Diagnosed by pericardiocentesis and requiring treatment
Cardiac tamponade	Diagnosed by echocardiography and requiring treatment
Respiratory Complications	
Pneumonia	X-ray or CT confirmed and necessitating antibiotic treatment
Airway necrosis or fistulae	Confirmed by endoscopy
Respiratory failure	Need for mechanical ventilation for greater than 24 h in a patient who requires reintubation after surgery OR need for mechanical ventilation for greater than 72 h in a patient who is not extubated on the day of surgery [1]
ARDS	Berlin definition [2]
Atelectasis	X-ray or CT confirmed and requiring bronchoscopy
Air leak	Chest tube maintenance for air leak for > 7 days postoperatively
Pulmonary embolus	Confirmed by angio-CT scan
Pleural effusions	X-ray or CT confirmed and requiring treatment
Chest infection	Supported by positive bacterial culture
Pneumothorax	X-ray or CT confirmed and requiring treatment
Mediastinitis	Supported by positive bacterial culture
Gastrointestinal complications	
Anastomotic leak	Extravasation of water-soluble contrast during a swallow study, visualization of either anastomotic dehiscence or fistulae during endoscopy or visible loss of saliva or methylene blue through the cervical wound
Nonanastomotic leak	Radiologically or endoscopically identified
Conduit necrosis	Endoscopically or intraoperatively identified
Intra-abdominal abscess	Supported by positive bacterial culture
Peritoneal effusion	Doppler ultrasound or CT confirmed and requiring treatment
Gastrointestinal bleeding	Requiring intervention or transfusion
Liver failure	Need for FFP to correct INR in patient with serum bilirubin > 12 mg/dL OR INR > 2.5 in a patient with serum bilirubin > 12 mg/dL[1]
Intestinal obstruction	Clinical symptoms, radiological confirmation and needing treatment
Delayed gastric emptying	Clinical symptoms, radiological confirmation and needing treatment
Diaphragmatic hernia	Diagnosed by barium swallow or CT scan and then confirmed intraoperatively
**Other complication**	
Transient ischemic attack	Defined according to American Heart Association/American Stroke Association expert consensus
Cerebral infarction	CT or MRI confirmed
Cerebral hemorrhage	CT or MRI confirmed
Urinary tract infection	Presence of microorganisms in the urine accompanied by one or more of the following: dysuria, urgency, loin pain, tenderness, pyrexia or pyuria
Urinary retention	Requiring reinsertion of urinary catheter
Acute renal insufficiency	Doubling of baseline creatinine
Renal failure	Need for dialysis in a patient not on dialysis preoperatively
Wound infection	Requiring opening of wound or antibiotics
Bacteremia	Supported by at least one blood culture positive for pathogenic organisms
Sepsis	Temperature > 38°C or < 36°C, heart rate > 90 beats/min, WBC count > 12000 cells/mL or < 4000 cells/mL, and bacteremia
Postoperative hemorrhage	Postoperative bleeding > 2000 mL or need for reoperation
Recurrent laryngeal nerve paralysis	Identified by endoscopic examination
Chylothorax	Increase in chest tube output with enteral alimentation, change in the nature of the output to a milky appearance, and confirmation by a physical-chemical analysis of the fluid
Deep venous thrombosis	Clinical symptoms and vascular color echo-Doppler diagnosis
Fat necrosis	Clinical symptoms and exception of infection
Subcutaneous effusion	Clinical symptoms and requiring treatment

Abbreviations: ECG = electrocardiogram; CT = computed tomography; ARDS = acute respiratory distress syndrome; FFP = fresh frozen plasma; INR = international normalized ratio; MRI = magnetic resonance imaging.

REFERENCES

1. Strasberg SM, Linehan DC, Hawkins WG. The accordion severity grading system

of surgical complications. Ann Surg. 2009;250(2):177-86.

2. Ranieri VM, Rubenfeld GD, Thompson BT, Ferguson ND, Caldwell E, Fan E, et al. Acute respiratory distress syndrome: the Berlin Definition. JAMA. 2012;307(23):2526-33.
